# Editorial: Physical activity and cancer-associated cognitive change

**DOI:** 10.3389/fcogn.2024.1523982

**Published:** 2024-11-28

**Authors:** Diane K. Ehlers, Linda Trinh, Sheri J. Hartman

**Affiliations:** ^1^Department of Quantitative Health Sciences, Mayo Clinic – Arizona, Phoenix, AZ, United States; ^2^Faculty of Kinesiology and Physical Education, University of Toronto, Toronto, ON, Canada; ^3^Herbert Wertheim School of Public Health, University of California, San Diego, La Jolla, CA, United States; ^4^University of California San Diego Moores Cancer Center, University of California, San Diego, La Jolla, CA, United States

**Keywords:** physical activity, exercise, cognitive function, cancer, brain health

Cognitive decline due to cancer and its treatments is increasingly recognized as a clinical priority amid its growing prevalence and impact on survivors' quality of life and long-term health (Dijkshoorn et al., 2021; Janelsins et al., 2017; Lange et al., 2019). Often characterized as changes in executive function, processing speed, attention, and memory processes (Ahles and Root, 2018; Fleming et al., 2023), studies estimate up to 78% of survivors experience cognitive impairment (Dijkshoorn et al., 2021). Neuroimaging studies have provided evidence of changes in brain volume, structural integrity, and functioning in regions and networks associated with these cognitive processes (Fleming et al., 2023; McDonald et al., 2013, 2012; Amidi and Wu, 2019). Further, clinical models have proposed a number of risk factors and mechanisms to explain cancer-associated cognitive decline (CACD), such as treatment regimen, sociodemographic factors, genetic factors, impaired neurogenesis, systemic inflammation, oxidative stress, lifestyle behaviors, and other biopsychosocial effects of cancer (e.g., fatigue, depressive symptoms) (Ahles and Root, 2018; Fleming et al., 2023). Despite extensive literature to characterize CACD, evidence-based strategies to manage CACD are limited.

Regular physical activity (including exercise) is an evidence-based strategy for ameliorating neurocognitive changes associated with normal aging (Northey et al., 2018). Among cancer survivors, evidence-based exercise guidelines are available for risk factors of CACD, such as cancer-related fatigue and depressive symptoms (Campbell et al., 2019). However, experts agree that, while data are promising, evidence remains *insufficient* to inform consensus guidelines regarding physical activity or exercise's cognitive benefits (Campbell et al., 2019; Sturgeon et al., 2023). To date, few fully-powered trials have been conducted (Witlox et al., 2019; Gentry et al., 2018; Hartman et al., 2021; Brunet et al., 2020), and findings have been mixed (Koevoets et al., 2022, 2023; Bender et al., 2024; Brunet et al., 2024). This remains a major gap in the literature, and, therefore, this Research Topic aimed to gather research to deepen our understanding of physical activity and CACD. Although none of the studies included were fully-powered, randomized controlled trials, they addressed other persistent knowledge gaps, including: (1) cognitive benefits of alternative modes of physical activity, (2) when and for whom physical activity may have the greatest cognitive benefit, and (3) neural mechanisms of cognitive improvement through physical activity.

This Research Topic illustrates that efforts to examine modes besides aerobic physical activity [which is most often investigated (Campbell et al., 2020)] are increasing, as three of the seven studies examined mind-body or meditative movement exercise interventions (*n* = 2 yoga, *n* = 1 Tai Chi and Qigong; mostly breast cancer survivors) (Neville et al.; Gothe et al.; James et al.). Results suggest mind-body exercise may provide a promising approach for improving self-reported cognition among cancer survivors. However, the extent to which mind-body exercise, especially lighter intensity approaches, can elicit meaningful improvements in cognitive performance is unclear, as limited to no benefit was observed in performance on cognitive tasks. Further, all three studies were preliminary or exploratory, delivered interventions of only 8–12 weeks in duration, and utilized heterogeneous methodologies to measure cognition. Consistent with previous systematic reviews of yoga interventions for mitigating CACD (Baydoun et al., 2020; Farahani et al., 2022), this reinforces the need for more rigorous trials in this area, in addition to common measurement approaches to improve homogeneity of methodologies.

Findings of the mind-body studies are similar to cross-sectional results in women prescribed chemotherapy and/or hormonal therapy for breast cancer as reported by Hartman et al.. Positive associations were observed between average daily minutes of moderate-to-vigorous physical activity and self-reported cognitive abilities, but there was no association with cognitive performance. These results are also consistent with data from early fully-powered trials in which investigators observed improvements in self-reported cognition in exercise groups compared to control, but no differential improvement in cognitive performance (Koevoets et al., 2022; Brunet et al., 2024). Despite limited primary findings, results from Hartman et al. provide insight on when and for whom physical activity may be most beneficial. Associations between physical activity and cognitive performance did not differ based upon treatments received (chemotherapy vs. hormonal therapy vs. both); however, greater physical activity was associated with better processing speed performance in survivors < 2 years from diagnosis. These findings are generally consistent with previous studies indicating exercise improves processing speed in subsets of breast cancer survivors, including those who are < 2 years from diagnosis (Hartman et al., 2018), prescribed endocrine therapy (Koevoets et al., 2022; Bender et al., 2024), or report high fatigue burden (Koevoets et al., 2022). Taken together, this evidence suggests self-report cognition and processing speed performance may be amenable to exercise in breast cancer survivorship. However, it is also possible these outcomes may be more sensitive to change and/or indicative of other health outcomes known to improve with exercise (e.g., depression, quality of life). As such, investigators should continue to utilize comprehensive batteries targeting multiple cognitive domains and consistent with International Cognition and Cancer Task Force (ICCTF) recommendations (Wefel et al., 2011).

Salerno et al. describe one of the few exercise programs designed to intervene upon cognitive function early in the cancer care continuum (Brunet et al., 2024), and results will contribute preliminary information on aerobic exercise during neoadjuvant or adjuvant chemotherapy for breast cancer, in addition to data on implementation of exercise programming during this period. This study may also provide insights into which patients may benefit most from an exercise intervention. Still, more research is needed, as these efforts represent very early progress on moderators (i.e., timing of intervention relative to cancer diagnosis and care) of exercise's cognitive benefits. There are myriad additional potential moderators proposed in the literature, spanning genetic, behavioral, functional, psychosocial, clinical, demographic, and contextual factors (Ahles and Root, 2018; Stenling et al., 2021; Erickson et al., 2019; Pesce et al., 2023).

Investigations of mechanisms of how physical activity can improve CACD are also necessary to advance the design of exercise and physical activity interventions to support cognitive health in cancer populations. Two studies in this Research Topic are among the few to examine neural outcomes associated with exercise in cancer survivors. Lesnovskaya et al. reported positive, cross-sectional relationships between baseline cardiorespiratory fitness and resting-state functional connectivity (rsFC) in breast cancer survivors enrolled in a randomized controlled exercise trial. Page et al., in a pilot randomized exercise trial in breast cancer survivors, observed small to moderate improvements in brain network modularity, a measure of functional brain organization and a particularly novel rsFC metric for investigation in exercise studies (Baniqued et al., 2018), among women randomized to 12 weeks of aerobic exercise. Importantly, these studies address neuroimaging outcomes recommended by ICCTF (Deprez et al., 2018); however, it is important to note the preliminary nature of the evidence and need for larger studies on exercise and brain structure and function (Koevoets et al., 2023). Further, consistent with the other studies in this Research Topic, no associations between exercise-related or neural metrics and cognitive performance were observed in either study.

Findings across studies raise additional considerations around conceptualization of self-reported cognition vs. objectively-measured cognitive performance. Both are important metrics, as previous research suggests self-report may tap into day-to-day cognitive performance (Lai et al., 2014) and recognizes the subjective experience and wellbeing of cancer survivors who report attention and concentration difficulties. However, it is critical that researchers studying the cognitive and neural impacts of physical activity acknowledge conceptual independence of cognitive function as measured by self-report vs. cognitive testing (Costa and Fardell, 2019). It is well-documented that self-reported cognition may have stronger association with psychological perceptions than with behavioral performance (Hanson et al., 2020; Collins et al., 2015). The effects of physical activity may also be different, and activity prescriptions with evidence of benefit for psychological function (e.g., depression, anxiety, mental fatigue) may more likely elicit improvements in self-reported cognition. In the present studies, authors failed to observe associations between activity-related outcomes and cognitive performance; (Hartman et al.; Lesnovskaya et al.; Page et al.) yet, associations with self-reported cognition were consistently observed. Further, improvements in self-reported anxiety and depression in breast cancer survivors were observed with Tai Chi or Qigong (along with improvements in self-reported cognition) (James et al.), and qualitative interviews with yoga participants indicated perceived benefits to mental wellbeing and stress (Neville et al.). These findings raise questions on whether the benefits observed reflect cognitive function, psychological function, or both.

In conclusion, while these studies demonstrate progress in the evidence base on physical activity and CACD, major gaps remain. More research with the following characteristics is needed: (1) high-quality trials with adequate sample sizes and long-term follow-up; (2) survivor populations other than breast cancer; (3) racially and ethnically diverse samples; (4) rigorous methodologies consistent with expert recommendations (Wefel et al., 2011; Deprez et al., 2018) and assessing cognitive function as a primary outcome; (5) trials that focus on physical activity and cognition earlier in the cancer care continuum; (6) tests of modalities beyond moderate-intensity aerobic exercise; (7) investigations of dose-response; (8) analyses of moderators and mechanisms driving relationships between physical activity and cognition; and (9) study designs that provide the opportunity to examine efficacy and effectiveness, while characterizing implementation in real-world settings (Stensland et al., 2022).
